# A Model to Predict In-Hospital Mortality in HIV/AIDS Patients with Pneumocystis Pneumonia in China: The Clinical Practice in Real World

**DOI:** 10.1155/2019/6057028

**Published:** 2019-02-17

**Authors:** Liang Wu, Zhe Zhang, Yu Wang, Yiwei Hao, Fang Wang, Guiju Gao, Di Yang, Jiang Xiao, Hongxin Zhao

**Affiliations:** ^1^Clinical and Research Center of Infectious Diseases, Beijing Ditan Hospital, Capital Medical University, China; ^2^The National Clinical Key Department of Infectious Diseases, Beijing Ditan Hospital, Capital Medical University, China; ^3^Division of Medical Records and Statistics, Beijing Ditan Hospital, Capital Medical University, China

## Abstract

We aimed to develop and validate a predictive model to evaluate in-hospital mortality risk in HIV/AIDS patients with PCP in China. 1001 HIV/AIDS patients with PCP admitted in the Beijing Ditan hospital from August 2009 to January 2018 were included in this study. Multivariate Cox proportional hazard model was used to identify independent risk factors of death, and a predictive model was devised based on risk factors. The overall in-hospital mortality was 17.3%. The patients were randomly assigned into derivation cohort (801cases) and validation cohort (200 cases) in 8:2 ratio, respectively, in which in derivation cohort we found that 7 predictors, including LDH >350U/L, HR>130 times/min, room air PaO_2_ <70mmHg, later admission to ICU, Anemia (HGB≤90g/L), CD4<50cells/ul, and development of a pneumothorax, were associated with poor prognosis in HIV/AIDS patients with PCP and were included in the predictive model. The model had excellent discrimination with AUC of 0.904 and 0.921 in derivation and validation cohort, respectively. The predicted scores were divided into two groups to assess the in-hospital mortality risk: low-risk group (0-11 points with mortality with 2.15-12.77%) and high-risk group (12-21 points with mortality with 38.78%-81.63%). The cumulative mortality rate also indicated significant difference between two groups with Kaplan-Meier curve (*p*<0.001). A predictive model to evaluate mortality in HIV/AIDS patients with PCP was constructed based on routine laboratory and clinical parameters, which may be a simple tool for physicians to assess the prognosis in HIV/AIDS patients with PCP in China.

## 1. Introduction

With the advent of era of antiretroviral therapy and widespread use of pneumocystis pneumonia (PCP) prophylaxis, the incidence of PCP has declined substantially in HIV/AIDS population. Buchacz et al. [1] reported that the incidence of PCP in HIV/AIDS population in European countries and US was less than 1 case per 100-person years. Currently, PCP occurred in patients with severe immunosuppression and those who were not aware of HIV status. Our previous study [2] also demonstrated that PCP was the most predominant opportunistic infection and prevalence was 22.4% in HIV-infected population without initiating antiretroviral therapy (ART) in China.

PCP was still a life-threatening opportunistic infection in HIV/AIDS patients with severe immunosuppression, which often needed mechanical ventilation, especially in patients with severe PCP. Although different guidelines [3, 4] were established to improve the principle for treatment outcome and prognosis in HIV/AIDS patients complicated with PCP, PCP was the most important cause of death in HIV/AIDS patients with severe immunosuppression.

Prior studies [5, 6] indicated that, in HIV/AIDS-associated PCP, the risk factors associated with mortality included low hemoglobin level, low partial pressure of oxygen breathing room air, presence of medical comorbidities, older age, and pulmonary Kaposi sarcoma. At least one predictive score model was developed to predict mortality in HIV/AIDS-associated PCP at illness presentation [6], which was constructed in patients in western industrialized countries and was not validated in Chinese patients. Little was done to develop a prediction score model to predict the prognosis of HIV/AIDS-associated PCP in China; the aim of the study was to construct a simple clinical scoring model to predict the risk of mortality of HIV/AIDS-associated PCP, which provided healthcare workers an easy-to-use tool to identify PCP patient with higher risk of mortality in China so that clinical intervention was allocated as soon as possible.

## 2. Methods

### 2.1. Ethical Consideration

This retrospective study was carried out in Beijing Ditan Hospital, Capital Medical University, and the study protocol was approved by the research ethics committee of hospital, which complied with principles of Declaration of Helsinki. All of clinical and laboratory data were used anonymously and written informed consent was not required due to retrospective study using deidentified clinical information.

### 2.2. Study Population

**Figure 1:**
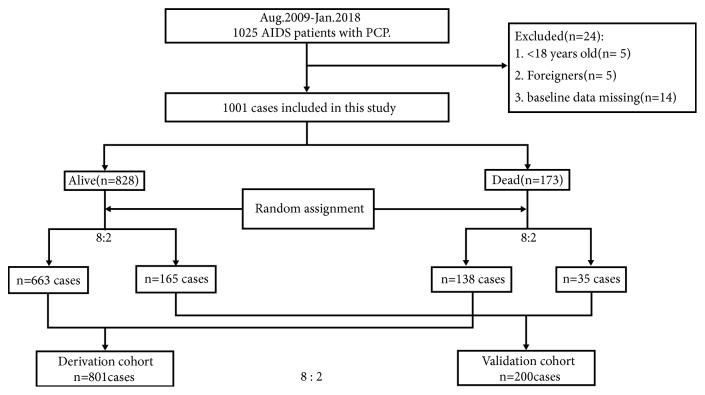
Flowchart of study population and random assignment with ratio of 8:2.

1025 AIDS patients with PCP who were admitted to Beijing Ditan Hospital, the national referral hospital for HIV/AIDS patients in China, were identified from August 2009 to January 2018 for retrospective analysis. Exclusion criteria were (1) aged < 18 years old; (2) overseas patients; (3) the patients who had baseline data missing (Figure 1). Clinical information was summarized from electronic medical records; in-hospital mortality was assessed after admission in these patients; and a clinical prediction model was developed to evaluate the prognosis and outcome of HIV/AIDS-associated PCP in China.

### 2.3. Diagnosis and Treatment

Diagnosis of PCP in HIV/AIDS patients was based on treatment guidelines of opportunistic infections (OIs) recommended by the National Institute of Health (NIH) of America [3]. HIV/AIDS patients had compatible clinical symptoms and signs such as dry cough, subfebrile temperature, heart rates, and dyspnea on exertion which were collected at the first presentation of interstitial pneumonitis when seeking medical attention performed bronchoalveolar lavage (BAL) as early as possible and a time lag between the time when patients were first seen at the hospital and that when a decision to perform bronchoscopy was made by treating or consulting clinicians was one day. Bronchoalveolar lavage fluid (BALF) samples were required to submit to laboratory for aetiologic diagnosis in respiratory system, including PCP. Cytopathologic demonstration of pathogen* pneumocystis jirovecii* in BALF samples was performed with Gomori methenamine silver which stained the cyst wall. Some patients with PCP with acute respiratory failure were not able to undergo bronchoscopy for confirmation after admission; these patients received empirical diagnosis and treatment; once symptoms and signs of acute respiratory failure were alleviated, bronchoscopic detection was performed as soon as possible.

Some other serum markers such as elevated (1,3)-ß-D-Glucan had a higher predictive value for diagnosis of PCP in HIV/AIDS patients [7], but in this study, diagnostic criteria of PCP were based on cytopathologic demonstration of pathogen* pneumocystis jirovecii* in BALF samples.

90% of the patients were ARV-naïve, and these patients were not aware of HIV infection until OIs became the first indicator of their disease. HIV infection was diagnosed when these patients sought medical attention. They were referred from other hospitals to Ditan hospital, the national referral hospital for HIV/AIDS patients in China. Patients with compatible clinical symptoms after admission were required to receive laboratory tests and perform computerized tomography (CT) scan and bronchoalveolar lavage (BAL) as early as possible, the time interval between presentation to the hospital and the clinical diagnosis of PCP was 1-2 days based on results of laboratory tests and CT scan, and time interval for definite diagnosis was 5-7 days based on cytopathologic demonstration of pathogen in BALF samples.

Diagnosis of OIs in HIV/AIDS patients was based on treatment guidelines of OIs recommended by the US National Institute of Health (NIH) [3], in which the diagnosis of cytomegalovirus (CMV) pneumonitis required consistent clinical and radiological findings (i.e., diffuse pulmonary interstitial infiltrates, fever, and cough or dyspnea), identification of multiple CMV inclusion bodies in lung tissue or cytology, and CMV DNA detection in bronchoalveolar lavage fluid (BALF) samples; and the diagnosis of fungal pneumonia was based on culture and identification of species of fungal pathogen with BALF samples or sputum.

PCP treatment was based on guidelines of OIs recommended by NIH [3] and trimethoprim/sulfamethoxazole (TMP/SMX) was the treatment of best choice. HIV/AIDS patients with moderate-to-severe PCP, which was defined as Alveolar-arterial O_2_ gradient >35mmHg or room air PaO_2_<70mmHg, initiated adjunctive corticosteroid treatment as soon as possible after initiating specific TMP/SMX therapy.

Highly active antiretroviral therapy (HAART) was recommended to HIV/AIDS patients with PCP based on National Free Antiretroviral Treatment Program (NFATP) in China [8], in which preferred regimen was tenofovir (TDF), lamivudine (3TC), and efavirenz (EFV).

### 2.4. Data Collection and Definitions

The social-demographic data included gender, age, and status of marriage, while clinical data included HIV transmission routes, breath and heart rates, duration of receiving ART, delayed admission to intensive care units (ICU), coinfected OIs with PCP patients, and documented comorbidities and complications. Laboratory data included baseline hemoglobin (HGB), albumin (ALB), CD4 cell counts, lactate dehydrogenase (LDH), and room air partial pressure of oxygen (PaO_2_) levels.

The prognosis recorded either survival or death when patients were discharged from hospital.

Severe pneumonia represented severe opportunistic infections in respiratory system with severe acute respiratory failure and/or septic shock due to immunosuppression in HIV/AIDS patient [9].

Delayed admission-to-ICU meant a hospital-to-ICU interval was more than 24 hours [10, 11]. The criteria for admission to ICU during the study period in Ditan Hospital were based on* guideline for ICU admission, discharge, and triage* recommended by US Society of Critical Care Medicine [12]; i.e., HIV/AIDS patients were critically ill and unstable, who required intensive monitoring or potentially needed immediate intervention which could not be provided outside of ICU.

### 2.5. Statistical Analysis

All data were analyzed using SPSS 20.0(SPSS Institute, Chicago IL, USA). The study population was randomly assigned as derivation and validation cohort, and clinical data were evaluated with percentages and chi-square test or Fisher exact test was used for statistical comparisons of these categorical variables between two cohorts.

Radom sampling was conducted using R software (3.3.3 version). 80% patients in alive and dead groups were randomly selected separately with random sampling procedure and were combined with derivation cohort, while other 20% in two groups were combined with validation cohort. Algorithm of randomization for the derivation and validation cohort we used was widely used in development of predictive score models [13, 14]

Cox proportional hazard models were used to evaluate the risk factors associated with mortality in HIV/AIDS patients with PCP in the derivation cohort. The integer scores were converted by rounding the hazard ratios (HRs) of the risk factors. For example, the HR of 1.860 associated with heart rate >130 times/min was equal to 2 points and the final score was the sum of these values. The prediction model was validated using the area under the receiver operating curve (ROC) curves in derivation and validation cohort. The maximum Youden index was determined based on ROC analysis. The correlation between prediction scoring model and mortality was plotted according to scores, and Kaplan-Meier survival curves were computed to compare difference in cumulative mortality between different groups.

In this study,* p *value of <0.05 was considered statistically significant.

## 3. Results

### 3.1. Patient Features

From August 2009 to January 2018, PCP was etiologically diagnosed in 1025 HIV/AIDS patients and 1001 patients were enrolled in this study based on exclusion criteria, in which 828 patients survived to discharge from hospital while 173 in-hospital death (17.3%) were found in the cohort. Random assignment in 8:2 ratio was conducted in survival group (663 &165 patients) and dead group (138 & 35 patients), respectively, to form derivation cohort (n=801) and validation cohort (n=200) in 8:2 ratio (Figure 1). The social-demographic and clinical features of these patients were available in Table 1, and no significant difference was found between features in two cohorts.

### 3.2. Development of Predictive Model of Mortality in HIV/AIDS Patients with PCP

Univariate Cox proportional hazard models were used to find risk factors of mortality in HIV/AIDS patients with PCP in derivation cohort; the results indicated significant difference in following variables: age>50years, CD4<50cells/ul, HGB≤90g/L, LDH≤350U/L, hypoalbuminemia, room air PaO_2_ <70mmHg, breathing rate ≥30 times/min, heart rate ≥130 times/min, later admission to ICU, and some comorbidities, including bacterial pneumonia, CMV pneumonitis, fungal pneumonia, severe pneumonia, and pneumothorax. Multivariate Cox proportional hazard model using above variables indicated that CD4<50cells/ul (HR 1.844, 95%CI 1.022, 3.326, and p=0.042), HGB≤90g/L (HR 2.063, 95%CI 1.220, 3.490, and p=0.007), LDH≤350U/L (HR 2.128, 95%CI 1.382, 3.279, and p=0.001), room air PaO_2_ <70mmHg (HR 7.328, 95%CI 3.621,14.830, and p<0.001), heart rate ≥130 times/min (HR 1.860, 95%CI 1.131, 3.060, and p=0.015), later admission to ICU (HR 6.418, 95%CI 4.212,9.781, and p<0.001), and pneumothorax (HR 1.630, 95%CI 1.027,2.588, and p=0.038) were independent predictors of mortality in HIV/AIDS patients with PCP (see Table S1 & Table 2).

The total scores from above 7 risk factors in each patients in derivation cohort ranged from 0 to 21 (Table 2), and ROC analysis indicated that cut-off scores of 11 matched the maximum Youden index, and the sensitivity and specificity for mortality in HIV/AIDS patients with PCP were 71.7% and 92.8%, respectively.

Predictive scores were assigned to above 7 risk factors based on rounding the HRs (Table 2), and a clinical score of mortality of individual HIV/AIDS patients with PCP was calculated with following formula [13]:

(1) $$\begin{array}{c} \text{Prognositic score}=2\times \left[\;CD4<50cells/ul\;\right]\\ +2\times \left[\;\text{Anemia }\left(\;HGB<90g/L\;\right)\;\right]\\ +2\times \left[\;LDH\geq 350U/L\;\right]\\ +2\times \left[\;\text{heart rate}\geq 130\;\;times/min\;\right]\\ +7\times \left[\;room\;\;air\;\;{pO}_{2}<70mmHg\;\right]\\ +6\times \left[\;\text{delayed admission to ICU}\;\right]\\ +2\times \left[\;\text{pneumothorax}\;\right] \end{array}$$

### 3.3. Validation of Predictive Model of Mortality in HIV/AIDS Patients with PCP

The area under the ROC curves in the validation cohort was used to evaluate the potency of predictive model of mortality in HIV/AIDS patients with PCP, and results indicated that area under the ROC curves (AUC) was 0.921 (95% CI: 0.882–0.961) in validation cohort, and AUC was 0.904 (95% CI: 0.875–0.934) in derivation cohort, which was more than 0.7, suggesting the predictive model to be effective to predict in-hospital mortality in HIV/AIDS patients with PCP (Figure S1).

### 3.4. Correlation between the Predictive Model and In-Hospital Mortality in HIV/AIDS Patients with PCP

**Figure 2:**
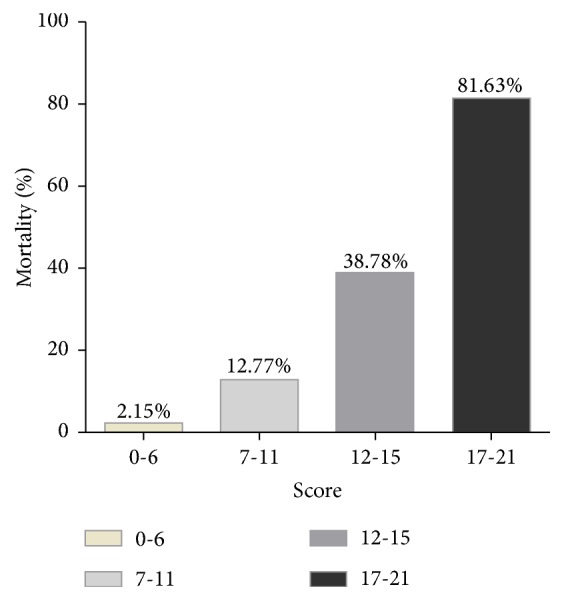
Percentage of mortality of HIV/AIDS patients with PCP according to the scores.* Note.* The integer scores were converted by rounding the hazard ratios of the predictors and the final score was the sum of these values.

**Figure 3:**
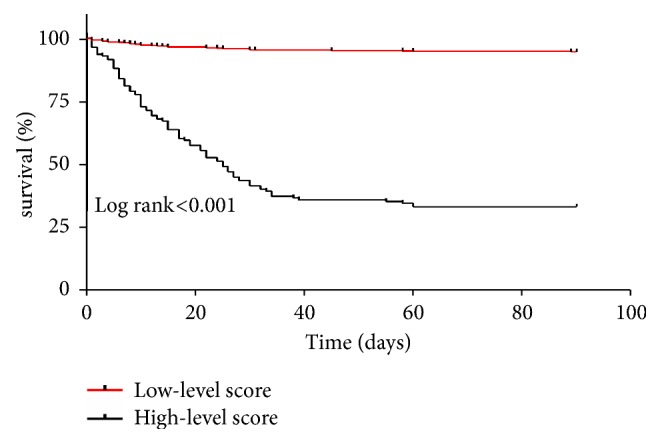
Kaplan-Meier survival curve of HIV/AIDS patients with PCP between groups of low-level and high-level scores.

The correlations between the predictive model and the in-hospital mortality in HIV/AIDS patients with PCP were further evaluated in this study, and results indicated that patients with higher scores showed poorer prognosis in our cohort. In-hospital mortality was 2.15% in patients with score of 0-6, 12.77% in patients with score of 7-11, 38.78% in patients with score of 12-15, and 81.63% in patients with score of 17-21, respectively (Figure 2). Cut-off scores of 11 matched the maximum Youden index, which indicated that total score 0-11 represented lower predictive risk and in-hospital mortality was 2.15-12.77%, while total score 12-21 represented higher predictive risk and in-hospital mortality was 38.78-81.63% (Table 2).  Figure 3 indicated that there was a significant difference in the cumulative in-hospital mortality between lower and higher predictive risk group in HIV/AIDS patients with PCP (*p*<0.001) (Figure 3).

### 3.5. Recurrent PCP, Trends of In-Hospital Mortality Rate, and Causes of Death during Study Period

TMP/SMX was the treatment of best choice for HIV/AIDS patients with PCP in China, recommended duration of therapy was 21 days, and PCP secondary prophylaxis was initiated immediately upon completion of therapy. HAART was recommended to HIV/AIDS patients with PCP once clinical symptoms were alleviated and temperature was normal. In this study, recurrent PCP was found in 20 patients based on second bronchoscopic detection and cytopathologic demonstration due to immune reconstitutional inflammatory syndromes after initiating antiretroviral therapy.

**Figure 4:**
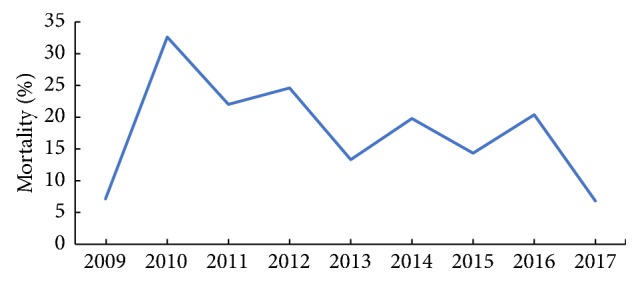
Trend of in-hospital mortality rates in study period.

In this study, 173 patients were dead in the hospital.  Figure 4 indicated a downward trend of in hospital mortality rates in study period.

In this study, 173 patients were dead in the hospital and the duration from admission to death was 14 days (6, 26.5) (mean value based on 25^th^, 75^th^ percentile), in which 137 patients were dead due to acute respiratory failure and the other 36 patients were due to infectious shock based on coinfected different opportunistic pathogens.

## 4. Discussion

The clinical management of AIDS-related opportunistic illnesses and ART for HIV infection had evolved significantly during study period. First, the time when to initiate ART was updated. Although antiretroviral regimens were freely provided to HIV/AIDS patients in China based on NFATP, the time when to initiate ART was updated from CD4 <200cells/ul to any CD4 cells levels, which reduced the incidence of OIs in China. Second, HIV testing and counseling were updated, which was changed from voluntary counseling and testing (VCT) to provider initiated testing and counseling (PITC) [15], which was beneficial to earlier find HIV-infected patients, especially patients with OIs, and reduced mortality. Third, various guidelines were updated [3, 4]. OIs were diagnosed and treated, and ART was initiated based on these updated guidelines, which affected the outcome of the study. Although rapid progress was made in HIV medicine, the diagnosis and management of OIs were an important challenge in China due to being unaware of HIV status in some HIV-infected patients. In this study, we aimed to develop a predictive model to evaluate in-hospital mortality risk in HIV/AIDS patients with PCP in China.

In this study, 90% of the patients were ART-naïve and HAART was recommended to HIV/AIDS patients with PCP as soon as possible once clinical symptoms were alleviated and temperature was normal based on National Free Antiretroviral Treatment Program (NFATP) in China [8], but there was some exception in interval from diagnosis of PCP to initiation of ART over the 10-year study period due to coinfection. In HIV/AIDS patients with PCP/tuberculosis coinfections and CD4<200cells/ul, ART was initiated within 2-4 weeks after starting TB treatment recommended by National Free Antiretroviral Treatment Program (NFATP) in China before 2016, while ART was initiated within 2 weeks after starting TB treatment in patients with CD4 <50cells/ul and within 2-8 weeks in patients with CD4 >50cells/ul recommended by US HAART guideline after 2016 [16]. Another exception for deferred initiation of HAART was PCP/cryptococcal meningitis coinfection in HIV/AIDS patients; ART initiation was deferred after 2-4 weeks of antifungal treatment with amphotericin B-containing regimens combined with flucytosine, or after 4-6 weeks of treatment with a high dose fluconazole recommended by WHO guideline in 2011 [17].

In this retrospective study of 1001 HIV-infected patients diagnosed as PCP in China, we found overall in-hospital mortality was 17.3%. The high mortality indicated importance of rapidly identifying patients with higher risk of death and earlier providing clinical intervention. In this study, we developed a clinically predictive model to assess prognosis in HIV/AIDS patients with PCP in China. In a multivariate Cox proportional hazard model, we found 7 predictors including LDH >350U/L, HR>130 times/min, room air PaO_2_ <70mmHg, later admission to ICU, Anemia (HGB≤90g/L), CD4<50cells/ul, and development of a pneumothorax were associated with poor prognosis in HIV/AIDS patients with PCP. A clinically predictive model to assess mortality was then developed based on above 7 predictors, which demonstrated that 38.78-81.63% of patients died in higher risk group and 2.15-12.77% in lower risk group.

Several studies [18, 19] have identified risk factors associated with poor prognosis in HIV/AIDS-associated with PCP, including prior receiving primary prophylaxis of PCP, lower hemoglobin and serum albumin, elevated LDH levels, the need for mechanical ventilation, development of a pneumothorax, and presence of comorbidities. These results were not consistent, because not all of studies demonstrated these risk factors were associated with HIV/AIDS-associated with PCP. In addition, these studies were conducted prior to introduction of antiretroviral therapy in western industrialized countries. Fei et al. [6] further developed a clinical score model, which indicated that age per 10-year increase, recent injection drug use, total bilirubin >0.6 mg/dl, serum albumin <3 g/dl, and alveolar-arterial oxygen gradient >or = 50 mm Hg were used to predict mortality from HIV/AIDS-associated PCP at illness presentation; this prediction score model was developed in United States but not validated in Chinese HIV/AID population with PCP, who had unique clinical features with Chinese characteristics.

Despite China's free ART program initiated in 2002 to save the lives and reduce the mortality of HIV/AIDS patients [20], OIs were the most important reason of morbidities and mortality in HIV/AIDS population in China [2]. Some patients developed severe OIs including PCP being not aware of their HIV status, and they sought medical assistance so late and their medical condition was serious. Some patients were aware of their HIV status but did not initiate ART due to social factors. Some patients presenting with an OI as the initial indicator of their complications started ART as an emergency response as soon as realizing their HIV infection, but short-term ART did not resulted in immediate immunological improvement.

Chen et al. [21] reported that cotrimoxazole prophylaxis administered earlier during ART reduced mortality in Chinese adults who were infected with HIV; Chinese free ART guideline also recommended cotrimoxazole prophylaxis in HIV-infected patients with CD4 cell counts less than 200 cells/ul [8]. Some patients did not receive cotrimoxazole prophylaxis due to several obstacles in China, including being unaware of their HIV status, poor knowledge of prophylaxis among healthcare workers, and poor adherence of patients.

Prior studies [22] indicated that OIs continued to cause morbidities and mortality in HIV/AIDS patients in China. In patients with severe immunosuppression, the same opportunistic pathogen can coinfect different organs, such as CMV, which can cause disseminated or localized end-organ diseases, including retinitis, pneumonitis, colitis, and neurologic diseases; and the different opportunistic pathogens can coinfect same organs, such as in respiratory system; besides* pneumocystis jirovecii, *other common opportunistic pathogens may include* Mycobacterium Tuberculosis, *CMV,* Candidiasis, Cryptococcus neoformans*, and* Aspergillus. *Coinfected opportunistic pathogens increased degree of difficulty of etiologic diagnosis in clinical practice, which was main reason for clinical deterioration and even death in patients with HIV-associated OIs in China.

Previous studies [23] indicated that CD4 cell counts <50cells/ul was associated with poorer prognosis in HIV/AIDS patients with OIs, including PCP, which was similar to our conclusion in this study, which indicated that monitoring CD4 levels in HIV/AIDS patients with PCP helped identify patients with increased risk of poor prognosis. It was undisputable that long-term HAART improve immune function in HIV/AIDS patients, but in this study, 89.7% of patients did not initiate HAART prior to admission due to being unaware of their HIV status, and as for other 8.6% receiving HAART for less than 6 months, we did not find that short-term HAART helped immunological improvement, which indicated that timely diagnosis of HIV infection and earlier receiving HAART may help reduce mortality in HIV/AIDS patients.

Anemia can result in abnormal physiological functions in ordinary population. It was also reported [24] that anemia was a predictor of disease progression and mortality in HIV/AIDS patients. Anemia often presented in HIV/AIDS patients with OIs, including PCP, which was treated as indicator of increased severity of OIs [25]. Walzer et al. [5] demonstrated that anemia was an independent predictor in HIV/AIDS patients with PCP. Moore et al. [26] indicated that correction of anemia reduced mortality and improved outcome of patients. Our results in this study indicated that anemia was an important risk factor of mortality in HIV/AIDS patients with PCP, which indicated that periodic screening routine blood test and timely correction of anemia were necessary in patients with PCP.

Serum LDH level was an unspecific marker for cell damage or death in path-physiological status, and in HIV/AIDS patients with PCP, elevated serum LDH level was associated with the degree of lung tissue damage [27]. In this study, our results indicated that serum LDH level >350U/L was an important risk factor of mortality in HIV/AIDS patients with PCP, which indicated severe damage of lung tissue, and periodic monitoring of serum LDH levels was important in these patients.

It was reported that [10, 11], in severe PCP in HIV/AIDS patients, earlier admission to ICU and receiving mechanical ventilation can improve prognosis in HAART era, and we previously found that [11] respiratory failure was the most common etiology in HIV/AIDS patients admitted to intensive care units, especially in patients with severe PCP; earlier admission to ICU was negatively associated with mortality, which was similar to our results in this study. The results in this study reminded physicians of being admitted to ICU earlier for patients with severe PCP, which may benefit from earlier receiving mechanical ventilation and prevent from clinical deterioration.

Development of pneumothorax was previously reported as a risk factor of mortality in HIV/AIDS patients with PCP [19]. Previous studies indicated that pneumothorax was a rare complication, and its incidence was 2%-9% in different cohort of AIDS-associated PCP [28, 29]. Pastores et al. [30] reported that abnormal lung remodelling during active PCP infection and rupture of preexisting parenchymal cysts or subpleural blebs likely contributed to the pathogenesis of pneumothorax. In this study, we found that pneumothorax was an independent risk factor of mortality and its incidence was 4% in HIV/AIDS patients with PCP in China, which indicated that pneumothorax was a fatal but rare complication and may be missing diagnosed or misdiagnosed in patients.

In this study, we found some risk factors that were amenable to interventions to affect the prognosis, which can be treated as a simple tool for physicians to assess the prognosis in HIV/AIDS patients with PCP. Besides risk factors we found, some other modifiable factors may also affect the prognosis of HIV/AIDS patients with PCP. Sun et al. [31] indicated that plasma IL-8, LDH, and HBDH levels and IL-6/IL-10 ratio could be helpful for early evaluation of the severity and predicting fatal outcomes in AIDS-associated PCP patients, which indicated that other modifiable risk factors of mortality should be further elucidated.

This predictive model had some strength. First, the clinical data collected were obtained from a large retrospective cohort over 9-year time period, which increased reliability of the conclusion. Second, the model was validated in a sizable validation cohort, which indicated high accuracy. Third, 7 predictors used in the model were routinely collected in clinical work, and simple calculation of scores was also conducive to implement clinical use of the model, which helped healthcare workers determine the prognosis and outcome of HIV/AIDS-associated PCP, especially in resource limited regions in China.

Besides these advantages, our study had some limitations. First, it was a retrospective study with inherent bias nature. Second, the study was conducted in a single center, and conclusion made in this study should be further validated in a prospective cohort. Third, the model was only used to evaluate the prognosis of patients who had confirmed diagnosis of PCP after admission; some patients who were too severe to confirm the diagnosis might be excluded in the study.

## 5. Conclusion

In conclusion, a predictive model to evaluate mortality in HIV/AIDS patients with PCP was constructed based on routine laboratory and clinical parameters, which may be a simple tool for physicians to assess the prognosis in HIV/AIDS patients with PCP in China.

## Figures and Tables

**Table 1:** Baseline characteristics of HIV/AIDS patient with PCP in the study cohort.

Variables	Total n=1001	Derivation cohort n=801	Validation cohort n=200	*p *value
*Age (years) *				
*⩾*50	188 (18.8%)	156 (19.5%)	32 (16.0%)	0.260
<50	813 (81.2%)	645 (80.5%)	168 (84.0%)	
*Gender*				
Female	77 (7.7%)	61 (7.6%)	16 (8.0%)	0.855
Male	924 (92.3%)	740 (92.4%)	184 (92.0%)	
*Transmission route*				
Homosexual	94 (9.4%)	71 (8.9%)	23 (11.5%)	0.484
Heterosexual	272 (27.2%)	218 (27.2%)	54 (27.0%)	
Blood transfusion	11 (1.1%)	7 (0.9%)	4 (2.0%)	
Intravenous drug	7 (0.7%)	6 (0.7%)	1 (0.5%)	
Unknown	617 (61.6%)	499 (62.3%)	118 (59.0%)	
*Marriage*				
Married	542 (54.1%)	439 (54.8%)	103 (51.5%)	0.401
Unmarried	459 (45.9%)	362 (45.3%)	97 (48.5%)	
*Laboratory results*				
HGB (g/L)				
Median (Q1, Q3)	120 (107, 133)	120 (107, 134)	119.7(107.3, 131)	0.403
>90g/L	918 (91.7%)	737 (92.0%)	181 (90.5%)	0.488
≦90g/L	83 (8.3%)	64 (8.0%)	19 (9.5%)	
ALB) (g/L)				
Median (Q1, Q3	31.8(28.3, 34.8)	31.7(28.3, 34.9)	32.1(28.5, 34.6)	0.539
>30g/L	646 (64.5%)	519 (64.8%)	127 (63.5%)	0.732
⩽30g/L	355 (35.5%)	282 (35.2%)	73 (36.5%)	
CD4 (cells/ul)				
Median (Q1, Q3)	21 (10, 47)	35.2 (10, 46)	41.6 (9, 53.8)	0.082
>50cells/ul	236 (23.6%)	183 (22.8%)	53 (26.5%)	0.276
≦50cells/ul	765 (76.4%)	618 (77.2%)	147 (73.5%)	
LDH (IU/L)				
Median (Q1, Q3)	334.1(252.8, 456.5)	336.8(251.4, 457.1)	321.8(259.2, 449.2)	0.198
*⩾*350 IU/L	456 (45.6%)	372 (46.4%)	84 (42.0%)	0.259
<350 IU/L	545 (54.4%)	429 (53.6%)	116 (58.0%)	
Partial pressure of oxygen				
>70mmHg	541 (54.0%)	434 (54.2%)	107 (53.5%)	0.863
⩽70mmHg	460 (46.0%)	367 (45.8%)	93 (46.5%)	
*Vital signs *				
Respiratory rate				
*⩾*30 times/min	142 (14.2%)	115 (14.4%)	27 (13.5%)	0.756
<30 times/min	859 (85.8%)	686 (85.6%)	173 (86.5%)	
Heart rate				
*⩾*130 times/min	58 (5.8%)	42 (5.2%)	16 (8.0%)	0.136
<130 times/min	943 (94.2%)	759 (94.8%)	184 (92.0%)	
*Duration of ART prior to admission*				
ART-naive	898 (89.7%)	720 (89.9%)	178 (89.0%)	0.618
<6 months	86 (8.6%)	69 (8.6%)	17 (8.5%)	
>6 months	17 (1.7%)	12 (1.5%)	5 (2.5%)	
*Later admission to ICU *				
Yes	183 (18.3%)	147 (18.4%)	36 (18.0%)	0.908
NO	818 (81.7%)	654 (81.6%)	164 (82.0%)	
*Comorbidities*				
Bacterial pneumonitis				
Yes	832 (83.1%)	663 (82.8%)	169 (84.5%)	0.559
NO	169 (16.9%)	138 (17.2%)	31 (15.5%)	
CMV pneumonitis				
Yes	385 (38.5%)	304 (38%)	81 (40.5%)	0.508
NO	616 (61.5%)	497 (62%)	119 (59.5%)	
Cryptococcal pneumonitis				
Yes	19 (1.9%)	17 (2.1%)	2 (1.0%)	0.298
NO	982 (98.1%)	784 (97.9%)	198 (99.0%)	
Fungal pneumonia				
Yes	209 (20.9%)	163 (20.3%)	46 (23.0%)	0.409
NO	792 (79.1%)	638 (79.7%)	154 (77.0%)	
Pulmonary tuberculosis				
Yes	148 (14.8%)	110 (13.7%)	38 (19.0%)	0.060
NO	853 (85.2%)	691 (86.3%)	162 (81.0%)	
Severe pneumonia				
Yes	148 (14.8%)	115 (14.4%)	33 (16.5%)	0.445
NO	853 (85.2%)	686 (85.6%)	167 (83.5%)	
Pneumothorax				
Yes	41 (4.0%)	34 (4.2%)	7 (3.5%)	0.635
NO	960 (96.0%)	767 (95.8%)	193 (96.5%)	
CNS infection				
Yes	54 (5.4%)	43 (5.4%)	11 (5.5%)	0.941
NO	947 (94.6%)	758 (94.6%)	189 (94.5%)	
Cardiovascular disease				
Yes	64 (6.4%)	46 (5.7%)	18 (9.0%)	0.092
NO	937 (93.6%)	755 (94.3%)	182 (91.0%)	
Malignancies				
Yes	27 (2.7%)	23 (2.9%)	4 (2.0%)	0.496
NO	974 (97.3%)	778 (97.1%)	196 (98.0%)	

*Note*. HGB: hemoglobin; ALB: albumin; LDH: lactate dehydrogenase; ART: antiretroviral therapy; ICU: intensive care unit; CMV: cytomegalovirus; CNS: central nervous system. Median (Q1, Q3): median based on 25th and 75th percentiles.

**Table 2:** Risk factors for mortality rate by Cox proportional hazard regression in HIV/AIDS patients with PCP and hazard rate and integer risk scores.

	Unadjusted HR (95% CI)	*p*-Value	Adjusted HR (95% CI)	*p*-Value	Predictive Score
CD4≤50cells/ul	2.860(1.612,5.072)	<0.001	1.844(1.022,3.326)	0.042	2
Anemia( HGB≤90g/L)	1.861 (1.118, 3.100)	0.017	2.063(1.220,3.490)	0.007	2
LDH≥350 IU/L	4.706(3.097,7.151)	<0.001	2.128(1.382,3.279)	0.001	2
Heart rate≥130 times/min	4.335(2.663,7.058)	<0.001	1.860(1.131,3.060)	0.015	2
PaO2≤70mmHg	19.193(9.748,37.789)	<0.001	7.328(3.621,14.830)	<0.001	7
Delayed admission to ICU	16.610(11.310,24.394)	<0.001	6.418(4.212,9.781)	<0.001	6
Pneumothorax	8.328 (5.397,12.850 )	<0.001	1.630(1.027,2.588)	0.038	2
*Overall risk level* *Mortality*					*Total score*
Low risk 2.15-12.77%					0-11
High risk 38.78%-81.63%					12-21

*Note*. HR: hazard ratio; CI: confidence interval; HGB: haemoglobin; LDH: lactate dehydrogenase; PaO2: partial pressure of oxygen; ICU: intensive care unit.

## Data Availability

The data used to support the findings of this study are available from the corresponding author upon request.

## Abbreviations

3TC: Lamivudine
ALB: Albumin
ART: Antiretroviral therapy
AUC: Area under the ROC curves
BAL: Bronchoalveolar lavage
BALF: Bronchoalveolar lavage fluid
CI: Confidence Interval
CMV: Cytomegalovirus
CNS: Central nervous system
CT: Computerized tomography
EFV: Efavirenz
HAART: Highly active antiretroviral therapy
HGB: Hemoglobin
HR: Hazard ratios
ICU: Intensive care unit
LDH: Lactate dehydrogenase
Median (Q1, Q3): Median based on 25th and 75th percentiles
NFATP: National Free Antiretroviral Treatment Program
NIH: National Institute of Health
OI: Opportunistic infection
PaO2: Partial pressure of oxygen
PITC: Provider initiated testing and counseling
PCP: Pneumocystis pneumonia
PTB: Pulmonary tuberculosis
ROC: Receiver operating curve
TDF: Tenofovir
TMP/SMZ: Trimethoprim/sulfamethoxazole
VCT: Voluntary counseling and testing.

## Supplementary Materials

Supplementary Materials
* Table S1: Risk factors for mortality rate by Cox proportional hazard regression in HIV/AIDS patients with PCP. Note*. CI: confidence Interval; HR: hazard ratios; HGB: haemoglobin; ALB: albumin; ART: antirretroviral therapy; PCP: pneumocystis pneumonia; CMV: cytomegalovirus; PaO2: partial pressure of oxygen; LDH: lactate dehydrogenase; CNS: central nervous system; PTB: pulmonary tuberculosis.* Figure S1. The area under the ROC curves (AUC) in the derivation cohort and validation cohorts.*
