# Supplementary material for: A Model to Predict In-Hospital Mortality in HIV/AIDS Patients with Pneumocystis Pneumonia in China: The Clinical Practice in Real World
Source: Biomed Res Int. 2019 Feb 17;2019:6057028. doi: 10.1155/2019/6057028 (PMC6398076; doi:10.1155/2019/6057028)
Supplement: Supplementary Materials — Table S1: Risk factors for mortality rate by Cox proportional hazard regression in HIV/AIDS patients with PCP. Note. CI: confidence Interval; HR: hazard ratios; HGB: haemoglobin; ALB: albumin; ART: antirretroviral therapy; PCP: pneumocystis pneumonia; CMV: cytomegalovirus; PaO2: partial pressure of oxygen; LDH: lactate dehydrogenase; CNS: central nervous system; PTB: pulmonary tuberculosis. Figure S1. The area under the ROC curves (AUC) in the derivation cohort and validation cohorts. [file 6057028.f1.pdf]

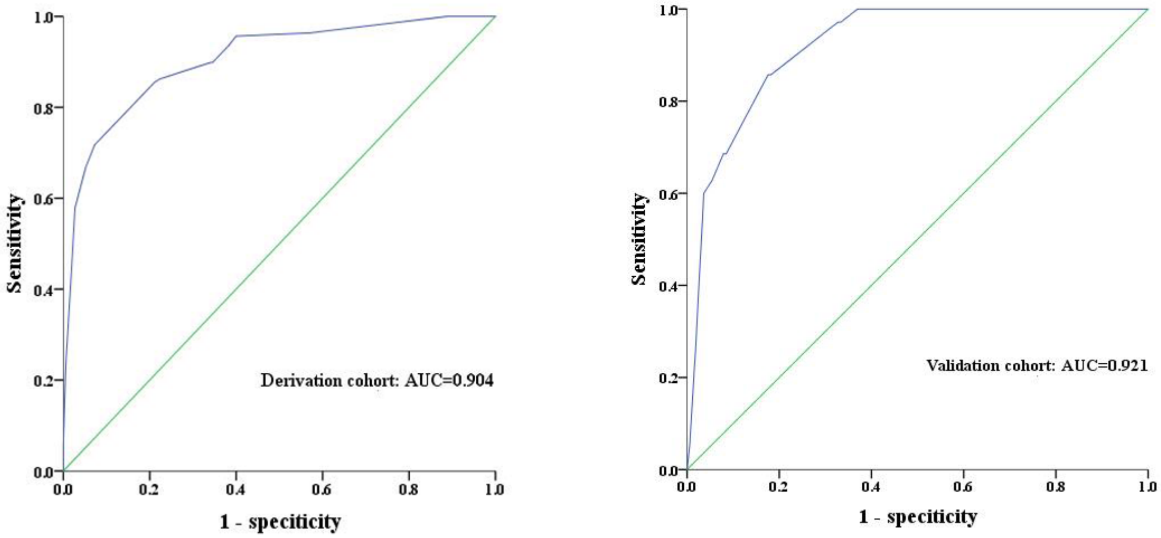

Figure S1. .The area under the ROC curves (AUC) in the derivation cohort and validation cohorts.

Table S1: Risk factors for mortality rate by Cox proportional hazard regression in HIV/AIDS patients with PCP.

Characteristics		Unadjusted HR (95% CI)	*p* value	Adjusted HR (95% CI)	*p* value
**Age (years)**	<50	1			
	≥50	1. 763(1.204,2.580)	0.004		
**Gender**	Female	1			
	Male	1.126(0.572,2.216)	0.731		
**Marriage**	Married	1			
	Unmarried	1.399(0.981,1.995)	0.063		
**Transmission route**	Homosexual	1			
	Heterosexual	0.977(0.441,2.166)	0.954		
	Blood transfusion	1.190(0.161,8.785)	0.864		
	Intravenous drug	4.681(1.413,15.507)	0.012		
	Unknown	1.729(1.112,2.688)	0.015		
**Laboratory results**	CD4＞50cells/ul	1		1	
	CD4⩽50cells/ul	2.860(1.612,5.072)	<0.001	1.844(1.022,3.326)	0.042
	HGB＞90g/L	1		1	
	HGB⩽90g/L	1.861 (1.118, 3.100)	0.017	2.063(1.220,3.490)	0.007
	LDH＜350 IU/L	1		1	
	LDH⩾350 IU/L	4.706(3.097,7.151)	<0.001	2.128(1.382,3.279)	0.001
	ALB＞30g/L	1			
	ALB⩽30g/L	1.899(1.346,2.678)	<0.001		
	PaO_2＞70mmHg	1		1	
	PaO_2⩽70mmHg				

		19.193(9.748,37.789)	<0.001	7.328(3.621,14.830)	<0.001
**Vital signs**	Respiration＜30 times/min	1			
	Respiration⩾30 times/min	0.238(0.166,0.341)	<0.001		
	Heart rate＜130 times/min	1		1	
	Heart rate⩾130 times/min	4.335(2.663,7.058)	<0.001	1.860(1.131,3.060)	0.015
**Duration of ART prior to admission**	＞6 months	1			
	＜6 months	1.086(0.599,1.967)	0.786		
	ART-naive	0.488(0.068,3.492)	0.475		
**Later addmission**	NO	1		1	
**to ICU**	Yes	16.610(11.310,24.394)	<0.001	6.418(4.212,9.781)	<0.001
**Comorbidity**					
Bacterial pneumonitis	NO	1			
	Yes	4.657 (2.053,10.567)	<0.001		
CMV pneumonitis	NO	1			
	Yes	1.606 (1.139,2.265)	0.007		
Cryptococcal pneumonitis	NO	1			
	Yes	1.117 (0.356,3.510)	0.849		
Fungal pneumonia	NO	1			
	Yes	1.729 (1.188,2.517)	0.004		
PTB	NO	1			
	Yes	0.751 (0.431,1.307)	0.311		
Severe pneumonia	NO	1			
	Yes	11.129 (7.846,15.787)	<0.001		
Pneumothorax	NO	1		1	
	Yes				
	NO				

CNS infection	Yes	8.328 (5.397,12.850)	<0.001	1.630(1.027,2.588)	0.038
	NO	1			
Cardiovascular disease	Yes	1.350(0.686, 2.657)	0.385		
	NO	1			
Malignancies	Yes	1.513 (0.816,2.806)	0.189		
		1			
		1.435 (0.587,3.509)	0.428		

Note: CI: Confidence Interval; HR: hazard ratios; HGB: Haemoglobin; ALB: Albumin; ART:antirretroviral therapy; PCP:pneumocystis pneumonia; CMV: cytomegalovirus; PaO_2: Partial pressure of oxygen; LDH: lactate dehydrogenase; CNS: central nervous system; PTB:pulmonary tuberculosis.
